# Patients’ Reported Reasons for Non-Use of an Internet-Based Patient-Provider Communication Service: Qualitative Interview Study

**DOI:** 10.2196/jmir.2683

**Published:** 2013-11-11

**Authors:** Cecilie Varsi, Deede Gammon, Torunn Wibe, Cornelia M Ruland

**Affiliations:** ^1^Center for Shared Decision Making and Collaborative Care ResearchOslo University HospitalOsloNorway; ^2^Norwegian Centre for Integrated Care and TelemedicineUniversity Hospital in North NorwayTromsøNorway; ^3^Abildsø Nursing HomeCenter for Development of Institutional Care ServicesOsloNorway; ^4^Faculty of MedicineUniversity of OsloOsloNorway

**Keywords:** communication, email, Internet, interviews as topic, patient dropouts, patient non-use, patient preference, professional-patient relations, qualitative research

## Abstract

**Background:**

The adoption of Internet-based patient–provider communication services (IPPC) in health care has been slow. Patients want electronic communication, and the quality of health care can be improved by offering such IPPCs. However, the rate of enrollment in such services remains low, and the reasons for this are unclear. Knowledge about the barriers to use is valuable during implementation of IPPCs in the health care services, and it can help timing, targeting, and tailoring IPPCs to different groups of patients.

**Objective:**

The goal of our study was to investigate patients’ views of an IPPC that they could use from home to pose questions to nurses and physicians at their treatment facility, and their reported reasons for non-use of the service.

**Methods:**

This qualitative study was based on individual interviews with 22 patients who signed up for, but did not use, the IPPC.

**Results:**

Patients appreciated the availability and the possibility of using the IPPC as needed, even if they did not use it. Their reported reasons for not using the IPPC fell into three main categories: (1) they felt that they did not need the IPPC and had sufficient access to information elsewhere, (2) they preferred other types of communication such as telephone or face-to-face contact, or (3) they were hindered by IPPC attributes such as login problems.

**Conclusions:**

Patients were satisfied with having the opportunity to send messages to health care providers through an IPPC, even if they did not use the service. IPPCs should be offered to the patients at an appropriate time in the illness trajectory, both when they need the service and when they are receptive to information about the service. A live demonstration of the IPPC at the point of enrollment might have increased its use.

**Trial Registration:**

ClinicalTrials.gov NCT00971139; http://clinicaltrial.gov/ct2/show/NCT00971139 (Archived by WebCite at http://www.webcitation.org/6KlOiYJrW).

## Introduction

A growing number of Internet-based patient-provider communication services (IPPC) are being offered to patients. These services provide patients the opportunity to have secure email contact with their health care providers over the Internet and can be a valuable supplement to traditional health services. An increasing number of studies indicate that IPPCs can help patients manage their illness better and improve health outcomes [1,2], improve patient centeredness [3], address unmet communication needs in health care [4,5], increase patients’ satisfaction [1,6-9], and improve quality of care [9-11]. Thus, better utilization of IPPCs is increasingly becoming part of health care policies [12].

Patients expect access to IPPCs in order to communicate with health care providers [9,13,14]. However, most studies have shown that only a small number of patients who are offered an IPPC actually make use of the service to communicate with their health care providers. A study from four ambulatory practices reported that 3.2% of the patients used an eVisit service [15]. A secure messaging system for diabetics was used by 19% of the patients [2]. An encrypted messaging system was used by 4.3% of parents with chronically ill children [16]. A secure messaging system in internal medicine was used by 31% of the patients [17], and two different secure messaging systems in primary care showed 6% use [18] and 52% use [19]. There is also evidence that patients are concerned about privacy and confidentiality [20,21], security [22], and trust [23], and many patients eventually stop using the service [24,25]. Use of email in the health care sector is not routine [26], even though the interest in using it is steadily increasing [27,28].

The IPPC in the current study is a further development of a secure multicomponent Web-based system for illness management support called WebChoice, which was designed to support cancer patients living at home between treatments and during rehabilitation. WebChoice allows cancer patients to monitor their symptoms and problems, provides individually tailored information and self-management support, and offers IPPC with cancer nurses as well as an e-forum for group discussion with other patients [29]. WebChoice has been tested in a randomized controlled trial (RCT) involving patients with breast or prostate cancer [30], where the patients were randomized into the WebChoice intervention group or a control group that received standard care. Results showed significant group differences in global symptom distress. In addition, patients in the WebChoice group had significant within-group reductions in depression. Self-efficacy and Health Related Quality of Life (HRQoL) significantly deteriorated in the control group, but not in the WebChoice group [30]. Interviews with 10 of the WebChoice users showed that some patients experienced the tool as highly supportive, while others had more ambivalent or conflicting feelings about it [31]; 38% of those who had access to WebChoice used the IPPC component where a study nurse responded to patients’ anonymous questions and concerns [32,33]. The IPPC was one of the components of the WebChoice package that patients spent most time using and was also the one they valued most highly [33].

A new study was initiated to determine whether the results from the RCT could be repeated when only the IPPC module was offered to the patients, without the other features of WebChoice. To utilize the full potential of the IPPC as a supplement to traditional health services, we also integrated the IPPC as a part of regular patient care into five hospital specialties and examined the implementation process simultaneously. To adapt the IPPC to patients’ needs and care providers’ requirements, we used several participatory methods in the design phase of the project, including research-practice networks, focus groups, workshops, heuristic evaluations, and usability testing [34]. Despite our efforts to make the service adaptable to the patients’ needs and desires, the IPPC was used by only 22% of the patients to whom it was offered—a participation rate significantly lower than in the previous WebChoice RCT, where patients could communicate anonymously with a study nurse.

This result led the research team to investigate the reasons for non-use in greater detail. A better understanding of user acceptance of IPPCs is essential in order to achieve effective implementation of such services into regular health care. The non-users of the service can provide valuable insights and explanations and thus contribute to the understanding of barriers to use and in the implementation process. We found that very little research has investigated non-use of IPPCs. One of the few studies that have examined patients’ reported reasons for non-use of an IPPC is a study among pediatric patients and their families [16]. Little is known about adult patients and their reasons for non-use of IPPC services. One research group investigated patients’ barriers to enrollment in a patient portal where one of the components was a secure electronic message system. They found that a lack of awareness of the patient portal or a lack of motivation were the primary barriers to enrollment [22]. Another research group conducted a survey among patients with no experience of e-consultations and found that the most prominent reasons for non-use were that the patients were not aware of the existence of the service, that they preferred to see a doctor, or that their doctor did not offer e-consultations [35].

A better understanding of why patients choose not to use IPPCs is needed for these services to be targeted and adapted to the patients’ needs and preferences and subsequently implemented successfully into health care. As argued by Eysenbach, studies of non-use will contribute to the understanding of impact and uptake of eHealth interventions, and therefore should be of great interest to researchers [24]. The aim of the current study was to identify the patients’ views of an IPPC and their reported reasons for non-use of the service.

In planning and designing the current study, we examined different models and frameworks suitable for implementation, adoption, and acceptance of new technology in health care [36-40]. These models focus on predicting use of the technology and on how people and organizations adopt and start using the services, while non-users, who are the focus of the current study, are given less attention. In addition, the models focus mainly on health care providers and their organizations and are sparser in the constructs that reflect the patients’ point of view. As no existing theory or framework was suitable, we did an open approach to obtain as much richness as possible during the study.

## Methods

### Study Design and Participants

This study is part of a larger study to examine the implementation of the WebChoice IPPC module as part of routine care in five hospital specialties. Participants were recruited at discharge from a hospital stay or an outpatient visit. To be eligible for the main study, participants had to be at least 18 years of age, be able to speak and read Norwegian, have secure Internet access at home using a public key solution for secure electronic identification, and have one of five diagnoses or treatments: (1) liver transplantation, (2) testicular cancer, (3) autologous stem cell transplantation, (4) advanced cancer and participating in a clinical drug trial, or (5) type 1 diabetes. For the current study, the additional inclusion criterion was non-use of the IPPC after they had enrolled in the study and had access to the service for at least 6 months. To get an in-depth understanding of the reasons why the patients did not use the service, we conducted a qualitative study [41] based on individual interviews with non-users of the service. The study was approved by the Regional Committee for Medical and Health Research Ethics and the Data Security Inspectorate in Norway. Written informed consent was obtained from all participants.

### Internet-Based Patient–Provider Communication Service

**Figure 1:**
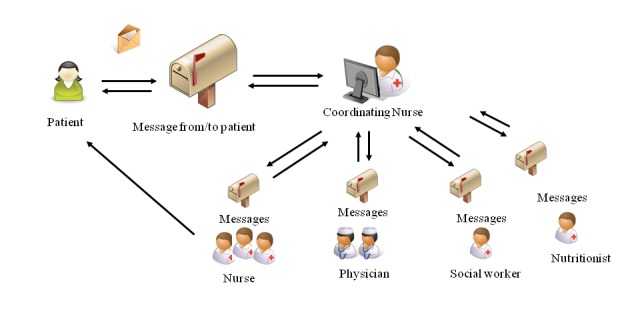
IPPC message flow between patients and health care providers.

The IPPC in this study is an Internet-based system where patients can send messages to and receive answers from hospital nurses, physicians, nutritionists, and social workers. The IPPC system has a high security level, requiring both patients and health care providers to log into the system by means of strong authentication keys. The IPPC is designed so that patients can get access to advice at the right level of expertise within the same system, without needing to know who the right person to ask is. The message from the patient is received in the mailbox of the coordinating nurse. In this study, the coordinating nurse had expertise on the respective diagnoses and treatments and had access to the patients’ medical record at the hospital. The nurse could address the question directly or forward the message to the mailbox of another provider who was in a better position to answer the question (see Figure 1).

### Recruitment to the Study

Patients who met the inclusion criteria were invited to participate in the IPPC study by a nurse from the relevant hospital unit. The patients who were interested in participating in the study were referred to a member of the project team who explained the purpose of the study, asked for consent, and filled out the necessary registration forms and baseline questionnaires. The patients also received a brief introduction with information about how to log into and use the IPPC. They were informed that they could send messages with questions and concerns related to their illness and would receive advice and support from hospital health care providers in between and after their hospital admissions. The patients were informed that they could use the IPPC as much as they wanted over the study period, which lasted for 6 months for the patients with diabetes and 8 months for the other patients.

### Recruitment to the Interviews

**Figure 2:**
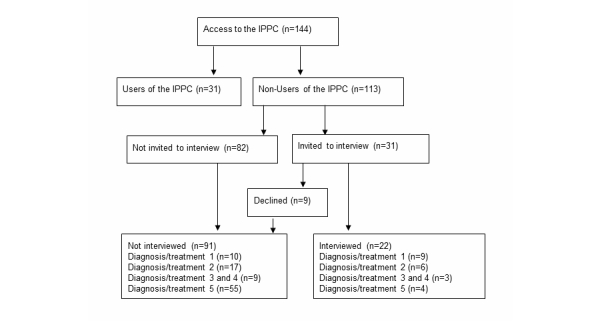
Patient recruitment for the interviews. Diagnosis/treatment 1: Liver transplantation. Diagnosis/treatment 2: Testicular cancer. Diagnosis/treatment 3: Autologous stem cell transplantation. Diagnosis/treatment 4: Advanced cancer and participation in clinical drug trials. Diagnosis/treatment 5: Type 1 diabetes.

We applied convenience sampling and asked some of the non-users who completed their study period either between January and November 2011, or in December 2012, to participate in an individual interview. Interviewees were selected from two different periods of time, to include patients from all five groups, as the study was conducted at different times at the different study sites. Criteria for participation in the interviews were that the patients had completed their study period. In addition, for practical reasons, they had to live within 180 km (110 miles) of the study hospital or have an appointment there. Due to the number of patients living far away, we subsequently also approved interviews over the telephone to achieve a sufficient number of interviews. There were 9 patients who declined participation while 22 patients agreed: 3 women and 19 men. The interviews were conducted by the first author either in a meeting room at the hospital, at the first author’s workplace, at the responder’s workplace, or by telephone. The interviews were recorded with a digital voice recorder and transcribed verbatim (see Figure 2).

### Measures

A semistructured interview guide based on literature on implementation and non-use of eHealth interventions [16,22,24,35] was developed to gather the non-users’ reasons for not using the IPPC. The interview guide contained five themes: (1) the diagnosis and treatment of the patients; (2) how the patients were introduced to the IPPC, their expectations of the service, and what they expected they could use it for; (3) reasons for non-use; (4) factors that could have influenced their use of IPPC; and (5) use of telephone, computers, and the Internet in their everyday life. Information about age was collected from the demographic form that they had completed at the point of inclusion to the main study.

### Analysis

The transcripts were analyzed using techniques of qualitative content analysis, inspired by a deductive directed approach, which is applicable when the analysis is investigating assumptions retrieved from prior research [42]. In the first step of the analysis, we identified variables as initial coding categories on the basis of the interview guide. Second, the first author coded the transcripts into the predefined categories using the framework approach of the software program NVivo version 9. Data that could not be coded into the predefined categories were given new categories. Next, the data were discussed within the research team, and the data were coded into the final list of categories. Not all the predefined categories were used, and new categories emerged. Additionally, some categories were combined.

The patients’ reasons for not using the IPPC, which fell into three main themes, are presented in the next section. In addition, the patients’ information needs, use of telephone, and the Internet in their daily life, as well as their views about the IPPC, are briefly presented.

## Results

### User Information

In total, 22 patients (19 men and 3 women) participated in the interviews. The patients were between 29 and 71 years old (mean 50, median 51.5). Nine had undergone a liver transplantation, 6 had been diagnosed with testicular cancer, 4 had type 1 diabetes, and 3 had either undergone an autologous stem cell transplantation or participated in a clinical drug trial.

Use of the computer was a daily activity for all the patients in the study, either related to their work or in their leisure time. They were neither novice nor expert users, but reported spending a moderate amount of time on the Internet. More than half of them had searched for health information on the Internet, most at the start of their illness, and the rest had not searched for health information on the Internet at all. Some of them said that they chose to use the telephone, text messages, or email on the basis of what they felt was most appropriate in the specific situation in their daily life. One of the patients said:

If I’m in a hurry I send a text message. If I want something confirmed, I’ll send an email. And if I have plenty of time, I’ll use the phone.

All of the patients with diabetes had received their diagnoses several years prior to the study. They reported being reconciled with the illness and said that it did not give them cause for concern in their daily life. The patients in the other four groups had in common that they had been quite ill during the disease trajectory, spent time in hospital, and had been through a convalescent period that had lasted for some time after discharge. They also had periods of sick leave before they could return to their ordinary daily lives. Only a few of them recovered quickly. Psychological reactions had also been a part of the illness. They worried about not getting well, about relapses of cancer, and about complications after treatment; sometimes they just had bad days or felt down.

Patients described anxiety before they knew what was wrong with them and felt reassured when they received information about the treatment and follow-up plan. Many of them had to wait for some time before receiving information, but when they got it, they felt that it was sufficient. They also reported being satisfied with the follow-up from health care providers during the hospitalization and after discharge. A few of them experienced that their primary care physician either was too busy or had too little knowledge about their particular situation to offer them sufficient follow-up.

Many of the patients viewed IPPC as a good tool for communication with health care providers at their hospital treatment unit. They felt it was a good service for them, and they were positive about the service. Some of them said that they liked having the opportunity to contact the hospital if they should feel a need for it and that they thought it could be helpful to many people. However, a few of the patients assumed right from the start, when introduced to the IPPC, that they would not use it.

### Reasons for Non-Use of the IPPC

#### Overview

The patients’ reported reasons for non-use of the IPPC were consolidated into three different explanations: (1) had sufficient access to information elsewhere, (2) preferred other types of communication, and (3) were prevented by conditions with the IPPC.

#### Had Sufficient Access to Information Elsewhere

Many of the patients explained their non-use of the IPPC with the fact that they did not have any questions, because they had long-term experience with their illness, they had already received in-depth information about their situation and how to take care of themselves, or they did not have any particular problems after discharge. One of them said:

I was given excellent information before I started the chemotherapy. I knew pretty much everything about how I would react and all that. So later on, when my reactions weren’t all that bad, I really haven’t had many problems.

The diabetes patients in the study had lived with their disease for many years. They reported that they had limited need of the additional support and follow-up offered by the IPPC since they were past the period in which they had many questions.

Those who had questions handled this in different ways. Patients said that they were used to finding answers to their questions on their own. Some talked to other patients in the same situation as themselves. The majority said they received information and could ask questions either in consultations with the primary care physician or at the hospital. Some said that their questions were not urgent, so they wrote lists of questions to bring to the next encounter. Some patients also reported having a close relationship with their physician, so that they could drop in if they had questions. Some of them also felt that the answers were given to them before they even thought of having any questions. One of the patients said:

Why I haven’t used it? It simply never occurred to me. I haven’t even called my doctor to ask about anything. I haven’t really wondered about anything in particular. So it has just never been an issue, it’s as simple as that. I feel like I’ve been given all the answers and all the information I need during my appointments. They took blood tests and X-rays and said everything looks fine.

Forgetting that the service was available after they returned home from the hospital was another explanation of non-use from many of the patients. A few of the patients reported having too much to think about in the first period after discharge, and some others did not have any questions during the first period at home. When the questions arose at a later point in time, they had forgotten about the service. Lack of time or of motivation was also reported as reasons for non-use. Some patients said that they did not prioritize logging into the IPPC when they had some time available.

#### Preferred Other Types of Communication

An often expressed reason for non-use of the IPPC was that the patients preferred to talk to the health care providers, either by telephone or in person, and their experience of getting answers quickly enhanced this practice. They also appreciated the opportunity to ask follow-up questions and wanted the health care provider to have that opportunity as well. Even if their question was not an urgent matter, they preferred getting an answer immediately. One patient explained:

When a question comes to my mind, I want an answer right away. It’s quite possible that I would have gotten an answer just as quickly that way [through the IPPC]. But there’s something special about talking to someone. I think that’s the most important thing.

One patient also said that the information seemed more trustworthy when it was explained verbally, instead of written. Some patients also stated that they had felt too ill to use the IPPC when they came home from the hospital:

When you’re a bit tired, how much can you do? Can you find enough energy to start your computer then? [...] It’s been like a part-time job, going to the doctor twice a week. [...] So I don’t have the energy to sit down with a research project, to put it plainly. I’m sure it would have been useful for me.

#### Prevented by Factors Associated With the IPPC

Not all the patients could remember the moment when they were introduced to the IPPC by the member of the project team. A few of the patients explained the lack of recall with the overwhelming amount of information they had received at the hospital, which had pushed the information about the IPPC into the background. Our study also revealed that not all patients had understood what the IPPC was, how the service was organized, and what they could use it for.

Some of the patients said it would have been more appropriate to introduce the IPPC at an earlier point of time in the disease trajectory. One patient said:

I clearly believe that such a service would certainly be perceived as positive for family, friends and especially the patient, in cases where the discovery of the disease is much closer in time than what is the case with me.

A few of the patients who wanted to use the IPPC, experienced login problems that hindered use. One of the patients said:

It was just fine right up until I tried to log in. That just didn’t work. [...] Everything crashed. Bam! and both my Internet connection and the program were gone. It was the same every single time. I typed in my password and the program disappeared.

Some of them also thought that the login procedure was too cumbersome and said that they had forgotten how to log into the IPPC. One of the patients tried unsuccessfully to get in touch with the study’s support service. This patient said that when one is ill, one lacks the energy to persevere, and it is therefore easy to give up.

Some patients also said that they did not rely on their questions reaching the correct provider, and therefore preferred to use the telephone. According to one patient:

It’s better if I call and get a clarification than if I send an email and then walk around wondering ‘has he received my email?’

Some of the study participants also felt an obligation to answer the questionnaires for the main study before it was fair for them to log into the IPPC. Since they lacked the energy to embark on the questionnaires, they also skipped logging in.

None of the patients expressed concerns about the system’s security level or concerns that unauthorized persons could get access to the messages in the IPPC. None reported not having the user’s manual. No one expressed concerns about bothering health care providers or reported that they felt their questions were too insignificant.

The patients in this study did not have many suggestions for what could have been done to encourage IPPC use. Most of them said they did not know, but some said they would have used it if they had not received sufficient follow-up from their health care providers, or if they had had more questions. One said that a reminder could have helped, and one said that better support with the login problems might have helped.

## Discussion

### Principal Findings

In this study, we investigated patients’ views of an Internet-based patient provider communication service that they could use from home for communication with their hospital health care providers and their reported reasons for non-use of the service. Our results show that the patients’ reasons for not using the IPPC can be divided into three main categories: they felt that they had sufficient access to information elsewhere, they preferred other types of communication, or they were prevented by conditions with the IPPC. But even if they did not use the service, they appreciated having the IPPC available. In the discussion of these finding, attentions is directed towards themes perceived as important by the patients and essential in relation to the design and operation of services such as IPPC in the future.

### Timing and Targeting

In the current study, the patients were told that they could use the IPPC as they wanted over the study period, with questions related to the disease that they were being treated for at the university hospital. Four of the five patient groups included in the study had recently been treated for serious cancer or liver diseases. They were under close follow-up with frequent encounters and thus had good opportunities to ask questions directly to the health care providers. At the point of inclusion they also were about to be transferred from the university hospital to the primary health care system and thus could have considered it more appropriate to direct their questions to the primary care providers and not to the university hospital where the IPPC was available. The fifth group in the study consisted of experienced diabetes patients who, at the time of inclusion, were past the period in which they had many questions. The current study also revealed that some of the patients felt too sick or exhausted to use the IPPC at the point in time when it was offered to them. This is consistent with other studies that found an association between poor health status and infrequent computer use [25,43]. Another of our earlier studies also found that different user characteristics are associated with different use patterns and that this is important to take into account in the targeting of Web-based support systems to patients with different characteristics [32].

The fact that some of the patients in the current study had not realized what the IPPC actually was, how they could use it, and for what purpose, may be due to information overload. As well as receiving information about the IPPC and the research study at the point of inclusion, the patients had to fill out many documents, and they had received substantial volumes of medical information from their health care providers. In addition, some of the patients said they did not feel in need of the service when they were introduced to it, but when problems or challenges arose later, they had forgotten about the service. Some patients also reported that they were too busy to start using the IPPC. Other studies have also found that this type of system can be forgotten [16,22] or that patients lack the time to use it [16].

Selection of the “wrong” users, that is, those who already are doing so well that they do not feel a need for the technology, is reported to prevent its use [44]. Such attrition can be seen as an indicator of a well-functioning health care service, where the patients get what they need through regular follow-up [44,45]. However, one might ask whether the patients have the sufficient insight into their own situation to evaluate how well they are actually doing [44]. In one of our other studies, we reported how a patient did not understand that his blood values indicated serious kidney failure, but when he used the IPPC this was discovered and action was taken to prevent permanent kidney damage [4]. This indicates that introduction of the IPPC must be targeted to the right patient groups [46] with an appropriate timing in the disease trajectory, both when the patients have questions, when they are capable of using the service, but also when they are receptive to information about the features available and the login procedures. This is consistent with the results in two of our other studies, where patients reported that they wanted the IPPC earlier in the disease trajectory [4,33].

### Privacy

Both the current study and earlier studies have shown that patients want to know who is reading their messages and that they want reassurance that the messages reach the right person [20,47]. In the current study, the patients did not know if the coordinating nurse would forward the message to another health care provider or if the message could be read by the whole team connected to the service. Others have also emphasized that patients may not be comfortable sending sensitive information to an office clerk [48]. This may have affected the patients’ use of the system and is a possible disadvantage with a triage-based system like this, at least compared to an anonymous service such as WebChoice, where the patients could communicate anonymously with a study nurse [30]. One can presume that patients will direct different types of questions to providers with whom they have already established a trusting relationship, as pointed out by Andreassen et al [23], from the questions they direct to providers who are completely anonymous. The health care providers’ knowledge of the patients and access to their medical record is an advantage in some respect, but it can also preclude the anonymity that was offered in the previous WebChoice study. This suggests that the solution one chooses can affect both use and types of questions posted, and that it seems important to take into account the patients’ requirement for privacy, anonymity, confidentiality, security, and trust in the design of IPPC services.

### Awareness

The results in this study are in line with other studies [22], showing that many of the patients did not use the IPPC because they did not feel a need for it at the time of enrollment. It has been suggested that reminders as “push” factors might encourage patients to use Internet-based interventions [44,45]. In the current study, we tested automated reminders on a small scale at one of the five study sites. The patients who had not made use of the IPPC after 1 or 3 months received a message encouraging them to use the IPPC. However, the non-use rate at this study site remained the same as at the other study sites.

Previous studies have found that patients prefer communication channels related to the type of question they have. Email is often preferred for simple interactions like refilling a prescription and general medical information whereas face-to-face encounters are preferred for more complex interactions like treatment instructions or communication about serious health issues [20,43,49]. However, some studies have also found IPPCs to be suitable for addressing serious concerns, questions, and unmet information needs [4,50]. Therefore, availability of a range of different communication channels is important to meet the different users’ needs.

In the current study, patients initiated all communication. Communication initiated by providers might have increased use. Personalizing messages from providers would be in line with proactive follow-up, and targeting and tailoring of messages can be used as a specific strategy for influencing health behaviors [45,46]. Reminders about the availability of the service, combined with disease-specific FAQs (Frequently Asked Questions) may be one way of facilitating meaningful use. However, it is essential to find the right balance between the independent user and the proactive team. In the current study, the access to the IPPC was offered in addition to regular follow-up. If the health care system wants to replace some face-to-face or telephone encounters with Internet-based communication, there is a need for robust organization of the service to ensure quality and safety, and a reimbursement policy must also be in place. There are many ways to utilize IPPCs, and development of comprehensive multifunction patient portals has been proposed because they might increase use [16]. Suggested areas for use are long-term illness management [51], medication management [52], personalizing treatment, tracking patients’ progress over time, communicating information about recovery after treatment, enhancing patient education, and giving patients the opportunity to express concerns and receive responses from health care providers [53]. One might think that the self-management activation in the latter services would increase use and satisfaction, but one study revealed that intensity rather than selecting the content of the service did matter [54]. However, many of the patients in the current study stated that they had no questions, so there is a need to ensure that artificial needs are not created. In our previous WebChoice study [33], patients recruited themselves by contacting the research group after receiving information about the study through newspaper advertisements, magazines, and websites, among others. They might thus have been more eager to use the system than the patients in the current study, where all patients fulfilling the inclusion criteria were asked to participate. Some of the patients in the current study said that they knew from the start that they did not want to use the IPPC; they just agreed to participate in order to be helpful and to support the research.

In terms of planning for studies like this, earlier studies have suggested that attrition needs to be taken into account and that Internet-based trials should plan for the worst case scenario of losing half of the participants during the first month of the intervention [45]. Non-use should be taken into account in the same manner.

### User-Friendliness

Some of the patients in the current study intended to use the IPPC, but when they tried to log in, they encountered obstacles and gave up. These patients reported having limited amounts of energy, so when they could not get into the system on the first try, many of them did not try again. Earlier studies have also shown how factors related to the systems structure and login procedures affect use [16,55]. In addition, it has been emphasized that a clear and shared understanding of the features available in the communication service is essential and that providing information at the time of enrollment can increase use of the system [22,56]. Other studies have found that lack of training is a barrier to use of email by patients with cancer [57] and that many people will not be able to make use of eHealth technologies without at the same time being offered support in how to use the services [58]. There is thus a need for IPPC systems to be reliable and for sufficient helpdesk services to be offered. Although computer skills are increasing in the population, electronic communication in health care is still new and unfamiliar to many patients. The patients who were offered the IPPC in the current study had experience in using computers, but they had no experience specifically with electronic communication with health care providers. They thus had limited background experience to guide the current use, and one may wonder whether they felt uncertain about deciding what types of questions to ask in the IPPC and what to bring to the face-to-face encounters. We suggest that demonstrating the IPPC live at a computer could be helpful and could increase understanding of the IPPC, empowering patients to make better-informed choices about use of the IPPC.

### Reassurance

Contacts with the health care system can be fragile. When patients have established good contact and communication with health care providers during consultations, the threshold for trying a new form of communication can be high, as patients do not know how it will turn out. Some of the patients in the current study said that they would have used the IPPC if the ordinary follow-up program had not worked so well. Findings from earlier studies indicated that patients did not feel a need for new forms of electronic communication when their clinic was responsive to their phone calls [16] and that some people found email more impersonal [16,20]. The patients in this study had different preferences, and some of them said that they would never make use of electronic communication with health care providers, but rather continue using the telephone and face-to-face consultations as before. To make use of the system, patients have to be convinced that the current system is better or can provide an add-on to the regular follow-up services.

Patients in this study did not make use of the IPPC, but they liked having the service available. This corresponds with other studies, in which patients reported being interested in using email to communicate with their health care providers, but their actual IPPC use was low [16]. Some of the patients in the current study stated that they viewed the IPPC as a back-up solution. They said they would have used it if their initial follow-up program had failed, for example, if they had not received satisfactory answers from their health care providers or if new questions or problems had arisen during the disease trajectory. Having the IPPC as an option can provide a sense of reassurance and be of value to patients, even if they choose not to use it. Non-use does not necessarily mean a lack of perceived benefit. Use is not a goal in itself, and the current study has revealed that the reasons why patients do not use the system are not always associated with the system features, but as much with the surrounding factors.

### Strengths and Limitations

There are a number of limitations to our study. Our data are qualitative, and we can obtain descriptions of the experiences of those included in the study but cannot statistically compare differences with other reported results. The study was conducted at a single university hospital, and the results may not be representative for other practice settings. Five different groups of patients were included to strengthen the transferability of the study, but four of the five groups consisted of severely ill patients who had recently undergone highly specialized life-saving treatment. These patients were thus quite different from, for example, chronically ill patients who represent many of the patients seeking health care. Three women and 19 men participated in the interviews. The results of the study might have been different if more women had participated. For example, women might bring a different perspective regarding fundamental aspects such as privacy, confidentiality, and security. The gender distribution in this sample reflected the gender distribution in the main study. Retrospectively, we see that women could have been oversampled in order to achieve a more even gender distribution for the interviews. A strength of the study is that the number of interviews was large enough to provide a variety of experiences and to allow sufficient depth in the analyses.

### Conclusion

This study offers insights into the reasons why patients who had access to an IPPC did not make use of it. Such knowledge is crucial for implementation of IPPCs to the health care service and can help timing, targeting, and tailoring of the IPPCs to different patient groups. Our findings indicate that patients like having the opportunity to send messages to health care providers through an IPPC, even if they do not make use of the service, and that they think it can be useful to many patients. There is need for more knowledge about how to reach those in need of an IPPC and to determine appropriate timing in the disease trajectory for introducing the service, when patients are receptive to information about how to use the service and for what purposes. Finally, we believe that demonstrating the service on a computer at the time of introduction could have increased the understanding of the service.

## Abbreviations

FAQs: frequently asked questions
HRQoL: health-related quality of life
IPPC: Internet-based patient-provider communication service
IT: information technology
RCT: randomized controlled trial
